# Reduction of the PI3K/Akt related signaling activities in skeletal muscle tissues involves insulin resistance in intrauterine growth restriction rats with catch-up growth

**DOI:** 10.1371/journal.pone.0216665

**Published:** 2019-05-09

**Authors:** Yan Xing, Jin Zhang, Hongling Wei, Hui Zhang, Yuhong Guan, Xinli Wang, Xiaomei Tong

**Affiliations:** 1 Department of Pediatrics, Peking University Third Hospital, Beijing, China; 2 Department of pediatrics, Beijing Jishuitan Hospital, Beijing, China; 3 Department of Pulmonary, Beijing Children’s Hospital, Capital Medical University, Beijing, China; Tohoku University, JAPAN

## Abstract

Individuals with intrauterine growth retardation (IUGR) are prone to insulin resistance, whereas the underlying molecular mechanisms remain unclear. In this study, we investigated if the PI3K/Akt signaling pathway in skeletal muscle tissues involves insulin resistance in IUGR offsprings, particularly ones with catch-up growth. An IUGR rat model was established by feeding rats an isocaloric (30.50 Kcal/g) diet containing 8% protein (low-protein diet) from day 1 of pregnancy until the birth of their pups. Glucometabolic parameters were measured and compared. Quantitative PCR and western blot were performed to assess mRNA and protein expression changes of the PI3K/Akt related signals in skeletal muscle tissues. Pearson analysis was used to assess the correlation of the PI3K/Akt signaling level and catch-up growth with the insulin resistance index (IRI). The values of fasting plasma glucose, fasting insulin and IRI were significantly higher, whereas insulin sensitivity index was significantly lower in IUGR offsprings than those in the controls. The PI3K mRNA and protein levels as well as the phospho-Akt^Ser473^ levels were significantly lower in IUGR offsprings compared to the controls. Reductions of GLUT4 as well as increases of PTEN and nuclear fractional PPARγ were detected in IUGR offsprings. Catch-up growth IUGR rats were positively correlated with insulin resistance and underwent more remarkable alterations of the PI3K, PTEN and GLUT4 expressions. Our results demonstrated that rats born IUGR developed insulin resistance later in life, which was likely mediated by reductions of the PI3K/Akt related signaling activities, particularly in those with excess catch-up growth.

## Introduction

Intrauterine growth retardation (IUGR), one of the major causes of perinatal mortality and serious complications [1,2], leads to increased predisposition to a variety of chronic diseases, such as hypertension/cardiovascular diseases, dyslipidemia, obesity, insulin resistance/diabetes, and other metabolic syndromes in adulthood [3,4]. It has been shown that insulin resistance might be the central pathogenesis of the above chronic metabolic diseases. Barker and Hales firstly proposed the “thrifty phenotype” hypothesis, which says that fetal under-nutrition is strongly associated with a number of chronic conditions later in life [5]. This increased susceptibility results from fetal metabolic and/or physiological adaptations [5]. This adaptation may cause permanent effects, such as reduction of pancreatic β-cells and insulin skeletal receptors, which results in insulin resistance later in life. However, the pathomechanisms of insulin resistance remain unclear in individuals with IUGR.

Insulin resistance is a state with a normal or higher insulin level but lower effect on the glucose uptake [6]. Insulin, a hormone produced in the pancreas by the islets of Langerhans, regulates the amount and/or concentrations of glucose in the blood [6]. Upon binding to its receptor, insulin facilitates glucose uptake into adipose tissue and skeletal muscle mainly through the glucose transporters such as GLUT4. In this process, a distinct signaling cascade including multiple enzymes involves, such as the phosphoinositide 3-kinase (PI3K)/Protein Kinase B (PKB, also known as Akt) pathway [7,8]. Any abnormalities in insulin signaling pathways may cause reduction of its sensitivity, as a result of insulin resistance.

Some experimental data revealed that IUGR or low birthweight is not the only cause leading to adult metabolic disorders [9,10]. In individuals with IUGR, catch-up growth during the early postnatal period also plays an important role in the development of insulin resistance, and a potential link between catch-up growth and insulin resistance has been reported [11]. Rapid catch-up growth IUGR had the highest mortality for coronary heart disease [9]. Furthermore, some studies highlighted that insulin resistance may be an early event by which catch-up growth IUGR predisposed to adult disease [12,13]. However, the effects of early catch-up growth during postnatal life on insulin sensitivity in individuals with IUGR and the potential molecular mechanisms are still elusive. In the present study, we investigated the dynamic changes of insulin sensitivity and the PI3K/Akt related signaling activities in skeletal muscle tissues as well as the correlation of catch-up growth with insulin resistance in rat IUGR offsprings.

## Materials and methods

### Animals and experimental designs

This study was carried out in strict accordance with the recommendations in the Guide for the Care and Use of Laboratory Animals of the Peking University. The protocol was approved by the Committee on the Ethics of Animal Experiments of the Peking University Third Hospital (*Protocol Number*: *LA2017200*). IUGR rat model was established according to the protocol described previously [14,15]. Sprague-Dawley rats, 8 weeks old, with body weights (BWs) 220–240 g, were purchased from Laboratory Animal Science Department of Peking University Health and Science Center (Beijing, China).

18 female rats were mated with males overnight and pregnancy was verified by examining the vaginal sperm plugs. Pregnant rats were *ad libitum* fed an isocaloric (30.50 Kcal/g) diet containing 8% protein (low-protein diet) or 20% protein diet (normal diet) from day 1 of pregnancy until the birth of their pups. Both diets were obtained from Beijing Huakangkang Biotechnology Co., Ltd. (Beijing, China). In this study, male pups were studied in order to avoid gender and hormonal influence. Both control and IUGR group had same litter size, 5 pups per litter. All pregnant rats during lactation and newborn rats after weaning at 21 days of age were fed the normal diet. Fresh diet and water were provided daily *ad libitum*. Diet assignment was performed by an animal technician who was not involved in the outcome assessments.

BWs were recorded at day 0 (birthweight), 2 days, 7 days, 14 days, and 21 days as well as 2 and 4 months of age after birth. Based on the birthweights, IUGR pups were defined as and confirmed by the lower birthweights minus 2 standard deviations (SD) than the controls. Based on the mean and SD of BWs, IUGR offsprings were further identified as i) with catch-up growth (CG-IUGR), ii) with over catch-up growth (OCG-IUGR), and iii) without catch-up growth (W/O CG-IUGR): BWs of the CG-IUGR group is between the “mean ± SD” of the BWs of the control group with the same sex and age; BWs of the OCG-IUGR group is higher than the “mean + SD” of the BWs of the control group with the same sex and age; BW of the W/O CG-IUGR group is less than the “mean—SD” of the BWs of the control group with the same sex and age [16]. To plot the growth rate, weight gained per day was calculated, and the fractional growth rate (weight gained per day, divided by birth weight; FGR) were analyzed from day 2 to 4 months [17].

Anesthesia was provided using isoflurane (2% inhalant), and all efforts were made to minimize suffering. 12 h after fasting, blood was collected from the angular vein of the control and the IUGR rats at day 21, month 2 and month 4 after birth, respectively. Plasma was separated, frozen in dry ice, and kept at -80°C until analysis. Rats were then sacrificed by cervical dislocation under anesthesia, and the skeletal muscle was dissected, weighted, snapped into liquid nitrogen, and stored at -80°C for further analysis.

### Measurement of glucose metabolic indices

The concentration of fasting plasma glucose (FPG) was measured by using the Accu-Chek Aviva meter (Roche, Shanghai, China). The concentration of fasting insulin (FINS) was determined by using the Mercodia Insulin ELISA Kit (cat. no. 10-1113-01; Mercodia, Uppsala, Sweden) according to the manufacturer’s procedure. Insulin sensitivity index (ISI) was calculated: ISI = 1/FPG (mmol·L^-1^) x FINS (mU·L^-1^). Homeostasis model assessment was used to calculate insulin resistance index (IRI): IRI = [FPG (mmol·L^-1^) x FINS (mU·L^-1^)]/22.5.

### Reverse transcription-quantitative polymerase chain reaction (RT-qPCR)

Total RNA was extracted by using Trizol reagent (cat. no. 15596026; Thermo Fisher Scientific, Inc., Wilmington, DE, USA). The concentrations of RNA were determined by using the ND-1000 spectrophotometer (NanoDrop Technologies; Thermo Fisher Scientific, Inc.). Totally, 2 μg RNA was reversely transcribed into complementary DNA (cDNA) by using the First-Strand cDNA Synthesis kit (cat. no. 11483188001; Sigma-Aldrich). qPCR was then performed on PE5700 Real-Time PCR system (Bio-Rad Laboratories, Inc., Hercules, CA, USA). The SYBR Green PCR Master Mix (cat. no. 1725270; Bio-Rad Laboratories, Inc.) was used, in which 2.0 μl of cDNA and 0.2 μM of the specific primer pairs (Table 1) were included. A two-step PCR program was executed: initial denaturation at 95°C for 10 min; 35 cycles of 95°C for 45 sec, annealing temperature (Table 1) for 45 sec and 72°C for 1 min. The mRNA expression levels of the *PI3K*, *Akt*, *GLUT4*, and *PTEN* were normalized to the housekeeping gene β-actin. The relative expression levels of the target genes were calculated by using the 2^-ΔΔCq^ method.

**Table 1:** Primer sequences employed for reverse transcription-quantitative polymerase chain reaction.

Gene	Primers	Annealing Tm	Product size
*PI3K*	F: 5’-aacacagaagaccaatactc-3’	52°C	195bp
	R: 5’-ttcgccatctaccactac-3’		
*GLUT4*	F: 5’-acccaccggcagcctctgatt-3’	58°C	132bp
	R: 5’ -ggtggcgtaggctggctgtt-3’		
*AKT*	F: 5’-gtggcaagatgtgtatgag-3’	51°C	195bp
	R: 5’ -ctggctgagtaggagaac-3’		
*PTEN*	F: 5’-aattcccagaggcgctatgt-3’	55°C	137bp
	R: 5’-gattgcaagttccgccactgaaca-3’		
*β-actin*	F: 5’-agcccagaacatcatccctg-3’	60°C	156bp
	R: 5’-caccaccttcttgatgtcatc-3’		

### Western blot assay

Total proteins were extracted from skeletal muscle tissues by using radioimmunoprecipitation assay buffer [25 mmol·L^-1^ Tris-HCl pH 7.4, 150 mmol·L^-1^ NaCl, 1% Nonidet-40, 0.1% SDS, 0.5% sodium deoxycholate (cat. no. D6750; Sigma-Aldrich, St. Louis, MO, USA)] supplemented with 1 mmol·L^-1^ Na_3_VO_4_ (cat. no. S6508; Sigma-Aldrich), 1 g·L^-1^ leupeptin (cat. no. L2884; Sigma-Aldrich) and 1 mmol·L^-1^ PMSF (cat. no. 93842; Sigma-Aldrich). Nuclear proteins were prepared by using the CelLytic NuCLEAR Extraction kit (cat. no. NXTRACT, Sigma-Aldrich). Protein concentrations were quantified by using the Pierce BCA Protein Assay Kit (cat. no. 23225; Thermo Fisher Scientific, Inc.). A total of 75 μg protein was separated on 8% SDS-PAGE, and then transferred to the Nitrocellulose Transfer Membrane (cat. no. ab133412; Abcam, Cambridge, MA, USA) by using the semi-dry transfer methods. The membrane was blocked at room temperature for 1 h in 5% bovine serum albumin prepared in tris-buffered saline containing 0.05% Tween-20 (TBST), and then incubated for overnight in cold room with the following primary antibodies: rabbit anti-phospho-Akt^Ser473^ monoclonal antibody (1:500; cat. no. 4058; Cell Signaling Technology, Inc., Danvers, MA, USA), rabbit anti-Akt polyclonal antibody (1:1,000; cat. no. 9272s; Cell Signaling Technology, Inc.), rabbit anti-PI3 Kinase p85 antibody (1:1,000; cat. no. 4292; Cell Signaling Technology, Inc.), rabbit anti-phospho-PI3 Kinase p85 (Tyr458)/p55 (Tyr199) antibody (1:1,000; cat. no. 4228; Cell Signaling Technology, Inc.), rabbit anti-GLUT4 polyclonal antibody (1:200; cat. no. ab654; Abcam), rabbit anti-PPARγ monoclonal antibody (1:1,000; cat. no. 2435s; Cell Signaling Technology, Inc.), rabbit anti-PTEN polyclonal antibody (1:500; cat. no. 9552; Cell Signaling Technology, Inc.), rabbit anti-histone H3 monoclonal antibody (1:1,000; cat. no. 9717; Cell Signaling Technology, Inc.), and rabbit anti-GAPDH monoclonal antibody (1: 10,000; cat. no. 5174S; Cell Signaling Technology, Inc.). Histone H3 and GAPDH was used as the internal reference, respectively. Following 3 washes with TBST, the membranes were incubated with horseradish peroxidase (HRP)-conjugated goat anti-rabbit IgG (1:10,000; cat. no. G-21234; Invitrogen; Thermo Fisher Scientific, Inc.) for 1 h at room temperature. Following 3 washes with TBST, the blots were developed by using the Enhanced Chemiluminescence Western Blotting Substrate (cat. no. 32109; Pierce; Thermo Fisher Scientific, Inc.), and the intensity of the bands was quantified by using the ImageJ software (version 1.51s; National Institute of Health, Bethesda, MD, USA).

### Statistical analysis

Results are presented as the mean ± SD. Birthweights were compared by using unpair *t* test. One-way and Two-way ANOVA analysis of variances followed by the Turkey’s post-hoc test was used to conduct statistical analysis among multiple groups at different time points. Pearson analysis was used for analyzing the association of the protein levels and the FGR with insulin resistance (SPSS 16.0, IBM Corporation, Armonk, NY). The value of *p < 0*.*05* was considered as statistical significance.

## Results

### IUGR rat model was established

**Fig 1:**
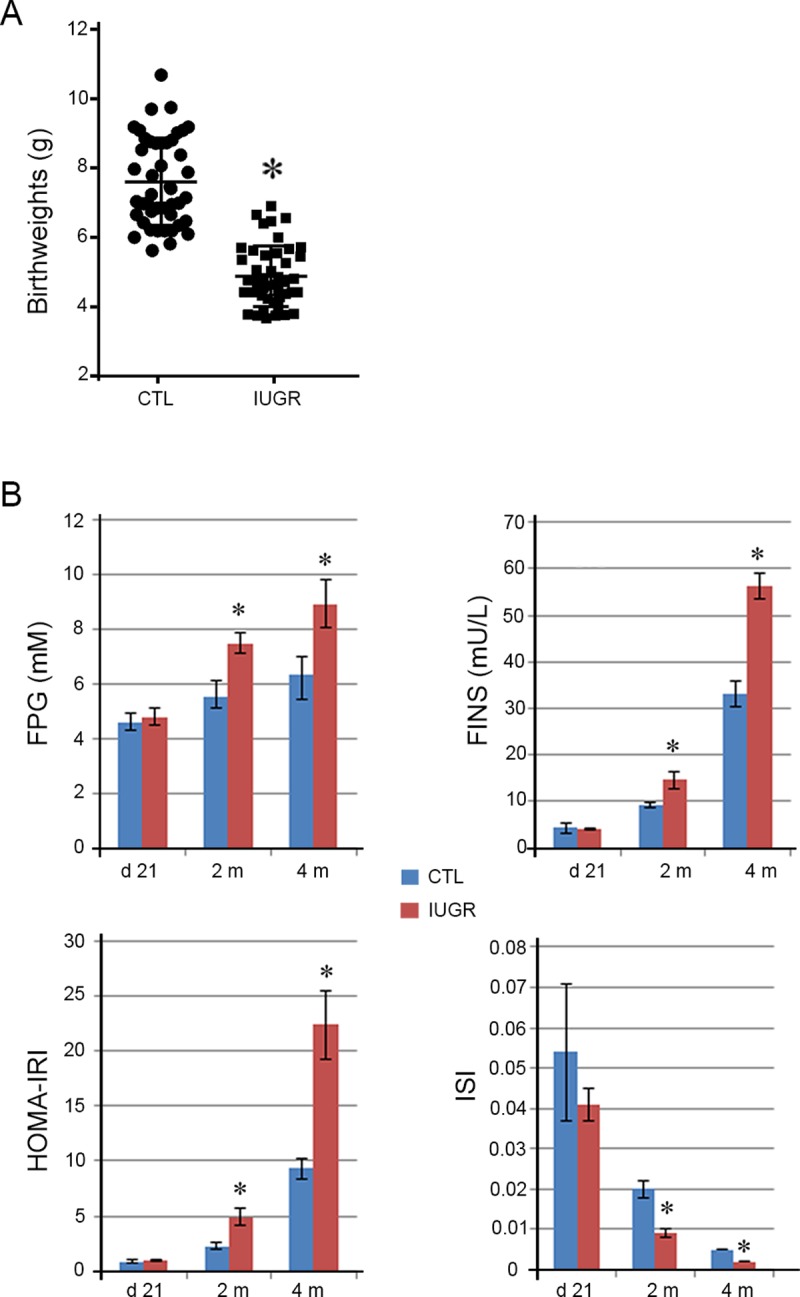
Insulin resistance was developed in the IUGR offsprings. A. Birthweights were shown for IUGR pups and the controls (CTL). The birthweights of the IUGR pups were significantly lower than those of the controls. *Data are shown as mean ± SD*. *n = 45*, ^*^p <0.*05 vs*. *CTL*. B. At 21 days, 2 months and 4 months of age, the concentrations of fasting plasma glucose (FPG) and fasting insulin (FINS) were as well as insulin resistance index (HOMA-IRI) and insulin sensitivity index (ISI) were compared between the IUGR offsprings and the controls. At day 21, the four indices did not show differences between the controls and the IUGR offsprings. At 2 and 4 months, the values of FPG, FINS and IRI were significantly higher, whereas the values of the ISI significantly lower in the IUGR offsprings than those in the controls. *Data are shown as mean ± SD*. *n = 15*, ^*^p <0.*05 vs*. *CTL*.

As described previously [14,15,18], IUGR pups were produced by feeding female rats the low-protein diet (8% protein) during gestation. The control group was fed normal diet (20% protein). The birthweights of the pups with IUGR were significantly lower (4.88 ± 0.94 g vs. 7.57 ± 1.20, *p* < 0.001) than those of the controls (Fig 1A). These results demonstrated that IUGR model was successfully established. The incidence of IUGR pups was ~93.33% in this study.

### Insulin resistance was developed in the IUGR offsprings

Insulin resistance is one of the major pathogenesis of diabetes. Some investigators showed that children with IUGR were predisposed to diabetes and other related metabolic diseases [3]. In our IUGR offsprings, we assessed glucose metabolic indices including FPG, FINS, HOMA-IRI, and ISI at 21 days, 2 months and 4 months of age after birth. Our results did not show significant differences of the four indices at day 21 between the IUGR offsprings and the controls (Fig 1B and Table 2). Nevertheless, the values of both FPG and FINS were significantly (*p < 0*.*001*) higher at 2 and 4 months in the IUGR offsprings than those in the controls. Further analysis displayed that the HOMA-IRI was significantly higher (*p < 0*.*001*) whereas the ISI was significantly lower (*p < 0*.*001*) at 2 and 4 months in the IUGR offsprings as compared to the controls. These findings implied that insulin resistance occurred later in the IUGR offsprings.

**Table 2:** Glucose metabolic indices in the controls and the IUGR offsprings.

	Day 21			2 months			4 months		
	CTL	IUGR	*p*	CTL	IUGR	*p*	CTL	IUGR	*p*
FPG (mM)	4.61 ± 0.34	4.79 ± 0.31	0.31	5.53 ± 0.58	7.49 ± 0.38	*< 0*.*001*	6.35 ± 0.66	8.94 ± 0.90	*< 0*.*001*
FINS (mU/L)	4.28 ± 1.04	4.21 ± 0.24	0.86	9.18 ± 0.66	14.67 ± 1.90	*< 0*.*001*	33.08 ± 2.76	56.33 ± 2.81	*< 0*.*001*
IRI	0.87 ± 0.21	0.90 ± 0.07	0.75	2.25 ± 0.31	4.90 ± 0.81	*< 0*.*001*	9.30 ± 0.90	22.44 ± 3.10	*< 0*.*001*
ISI	0.054 ± 0.017	0.041 ± 0.004	0.49	0.024 ± 0.002	0.009 ± 0.001	*< 0*.*001*	0.005 ± 0.000	0.002 ± 0.000	*< 0*.*001*

CTL: control, IUGR: offsprings with intrauterine growth restriction, FPG: fasting plasma glucose, FINS: fasting insulin, IRI: insulin resistance index, ISI: insulin sensitivity index.

### Dynamic alterations of the mRNA levels of PI3K, Akt, PTEN, and GLUT4 expressions in skeletal muscle tissues and their associations with insulin resistance in the IUGR offsprings

**Fig 2:**
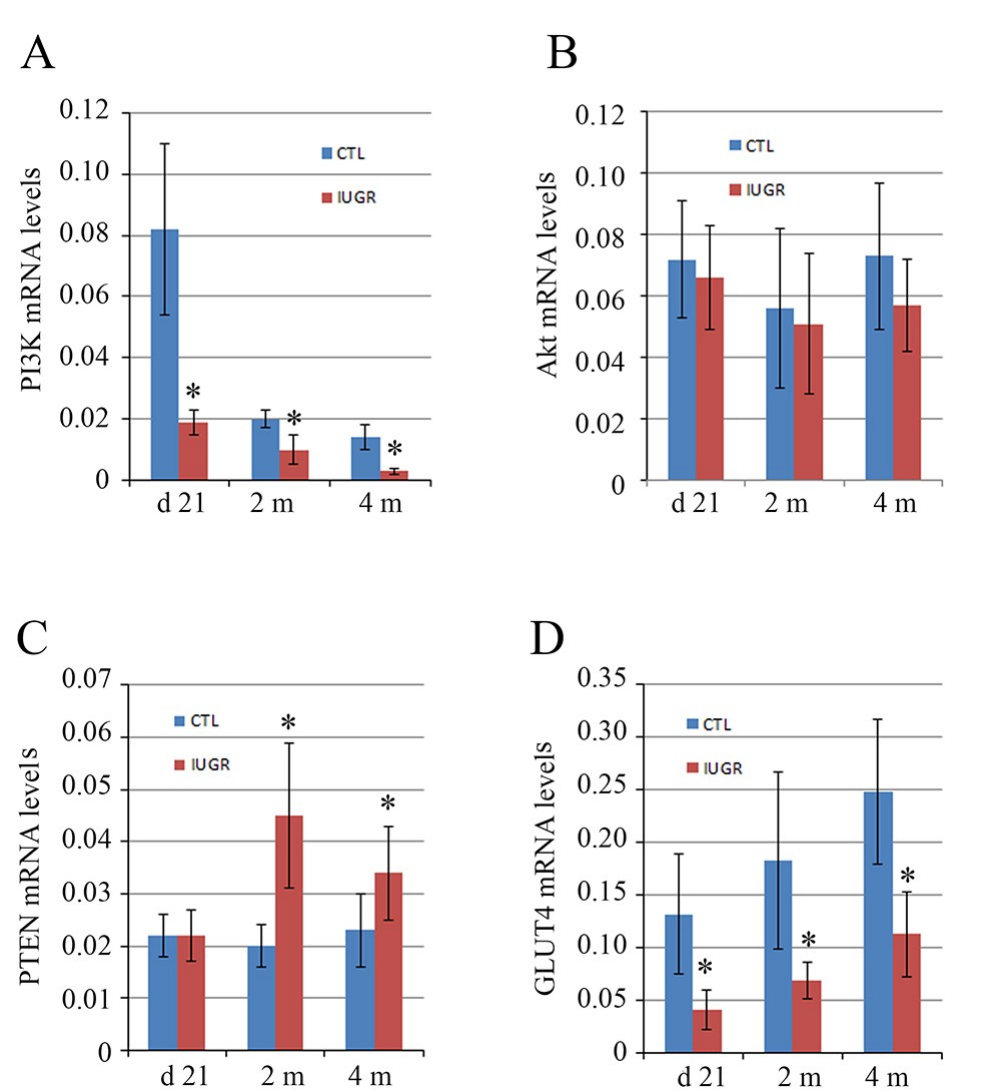
Dynamic changes of the mRNA levels of PI3K, Akt, PTEN and GLUT4 in the skeletal muscle tissue in the IUGR offsprings. The mRNA levels of PI3K (A), Akt (B), PTEN (C) and GLUT4 (D) were measured at 21 days, 2 months and 4 months of age after birth and compared between the IUGR offsprings and the controls (CTL). In comparison with the controls, the mRNA levels of PI3K and GLUT4 were significantly lower at all three time points in the IUGR offsprings. At 2 and 4 months, PTEN mRNA levels were higher in the IUGR offsprings than that in the controls. Akt mRNA levels did not show significant difference between the IUGR offsprings and the controls. *Data are shown as mean ± SD*. *n = 15*, ^*^p <0.*05 vs*. *CTL*.

**Fig 3:**
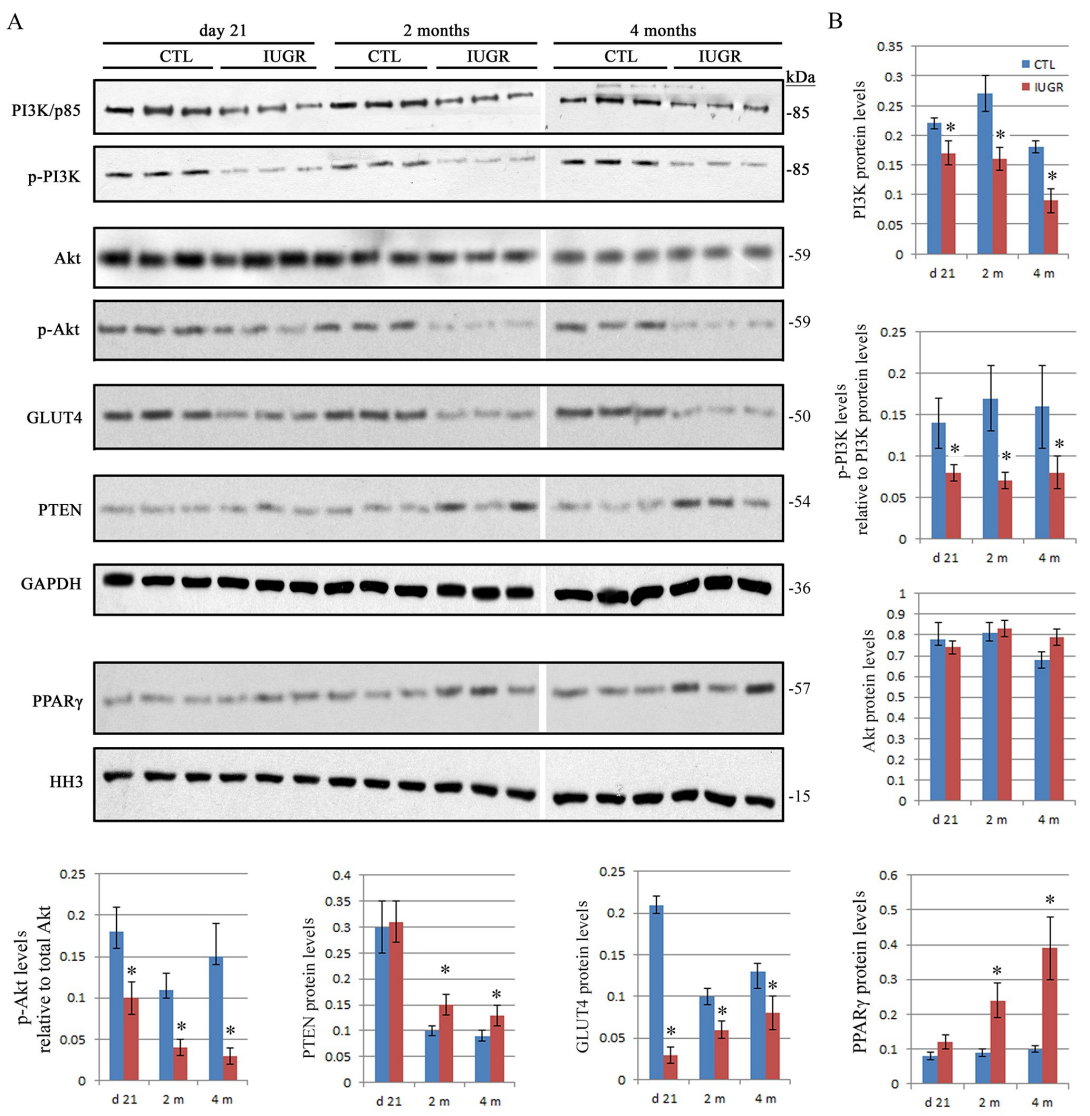
Dynamic changes of the protein levels of phospho-PI3K, phospho-Akt, PTEN, GLUT4 and PPARγ in skeletal muscle tissues of the IUGR offsprings. A. The representative western blot images are shown for PI3K, phospho-PI3K, Akt, phospho-Akt, PTEN and GLUT4 as well as the nuclear PPARγ. B. Graphed is the relative abundance of the total PI3K p85, Akt, PTEN and GLUT4 as well as PPARγ normalized to the housekeeping gene GAPDH and histone H3 (HH3), respectively. The levels of phospho-PI3K p85^Tyr458^/p55^Tyr199^, phospho-Akt^Ser473^ relative to the total PI3K p85 and Akt was compared, respectively. In comparison with the controls, the protein levels of total PI3K as well as the phospho-PI3K, phospho-Akt and GLUT4 were significantly lower at all three time points in the IUGR offsprings. At 2 and 4 months, the levels of PTEN and nuclear PPARγ were significantly higher in the IUGR offsprings than that in the controls. Total Akt did not show significant differences between the IUGR offsprings and the controls. *Data are shown as mean ± SD*. *n = 15*, ^*^p <0.*05 vs*. *CTL*.

Stimulation of insulin leads to a series of downstream signaling through binding to its receptors in target cells [19,20]. As a result, any defects or abnormalities of the insulin related signaling pathways may result in insulin resistance [21,22]. Currently, the PI3K/Akt pathway has been identified as one of the most important pathways associated with insulin resistance [23,24]. Here, we evaluated the abundances of the PI3K/Akt related proteins at the mRNA and protein levels in the IUGR offsprings. qPCR and western blot assays showed that the mRNA and protein levels of the PI3K were reduced significantly (*p < 0*.*05*) at day 21 as well as at 2 and 4 months in the IUGR offsprings in comparison with the controls (Figs 2A and 3). Total Akt did not show significant (*p > 0*.*05*) differences at the mRNA and protein levels between the IUGR offsprings and the controls (Figs 2B and 3), whereas the phosphorylated levels of both PI3K p85^Tyr458^/p55^Tyr199^ and Akt^Ser473^ were significantly (*p < 0*.*05*) downregulated at all three time points in the IUGR offsprings compared to the controls (Fig 3). These data suggested that the activation of the PI3K/Akt signaling was prohibited in the IUGR offsprings.

Phosphatase and tensin homology (PTEN) contains a tensin-like domain as well as a catalytic domain similar to that of the dual specificity protein tyrosine phosphatases [25]. Unlike most of the protein tyrosine phosphatases, PTEN preferentially dephosphorylates phosphoinositide substrates, and thus negatively regulates intracellular levels of phosphatidylinositol-3,4,5-trisphosphate in cells and functions as a tumor suppressor by negatively regulating Akt signaling [26]. Increased mRNA and protein levels of PTEN were significantly (*p < 0*.*05*) detected at 2 and 4 months in the IUGR offsprings as compared with the controls (Figs 2C and 3).

Glucose transporter type 4 (GLUT4), also known as solute carrier family 2, is a protein encoded, in humans, by the *SLC2A4* gene [27]. GLUT4, the insulin-regulated glucose transporter, is found primarily in adipose tissues and striated muscle, e.g. skeletal and cardiac [28]. Activation of Akt by a PI3K dependent mechanism stimulates translocation and activation of the GLUT4, and thereby induces glucose uptake [7,8]. The mRNA and protein levels of the GLUT4 were significantly (*p < 0*.*05*) reduced at day 21 as well as at 2 and 4 months in the IUGR offsprings in comparison with the controls (Figs 2D and 3). This provided further evidence of insulin resistance in the IUGR offsprings.

Peroxisome proliferator-activated receptor gamma (PPARγ), also known as the glitazone receptor, or NR1C3 (nuclear receptor subfamily 1, group C, member 3) is a type II nuclear receptor that in humans is encoded by the *PPARG* gene [29]. PPARγ has the highest expression levels in adipose tissue as compared with other metabolic organs, such as skeletal muscle, liver, and pancreas [30]. PPARγ is the master regulator of adipogenesis, thereby stimulating the production of insulin-sensitive adipocytes [31]. In addition, PPARγ activation in mature adipocytes induces expressions of a number of genes involved in the insulin signaling cascade, thereby improving insulin sensitivity [32]. Recently, the data in muscle-specific PPARγ deleted mice demonstrated that whole body insulin sensitivity is, at least in part, relying on the intact PPARγ system in skeletal muscle [33]. To assess the level of nuclear PPARγ, nuclear fraction was extracted and western blot assay was performed in the present study. The results revealed a significant (*p < 0*.*05*) increase of PPARγ protein in nuclear fractions at 2 and 4 months in the IUGR offsprings compared to the controls (Fig 3).

**Fig 4:**
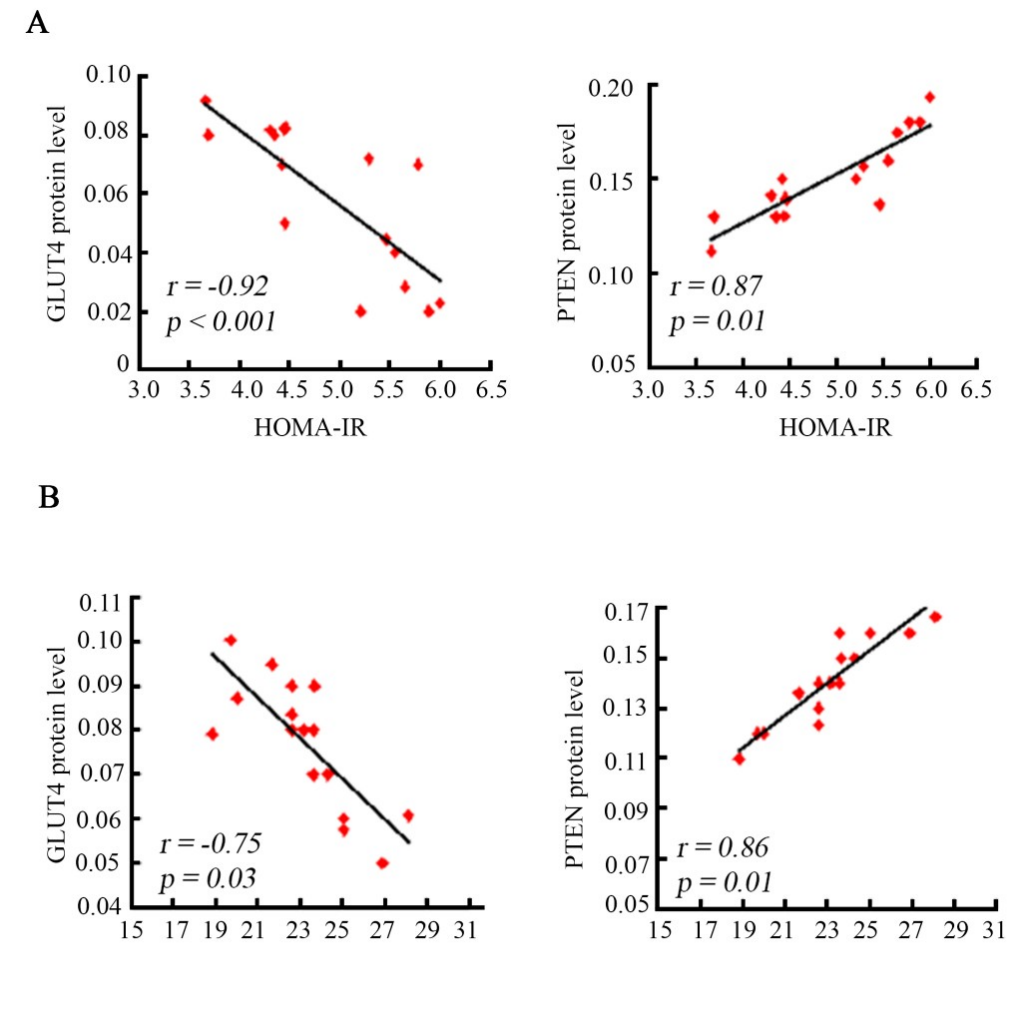
GLUT4 and PTEN protein levels were correlated with insulin resistance in the IUGR offsprings. Association analysis was conducted between GLUT4 or PTEN protein levels and the insulin resistance index (HOMA-IRI) at 2 (A) and 4 months (B) of age after birth in IUGR offsprings. The abundance of GLUT4 was negatively correlated with HOMA-IRI, and PTEN was positively correlated with HOMA-IRI.

To further analyze the relationship between the PI3K/Akt related signaling and the insulin resistance, we performed Pearson coefficient correlation analysis. Our results showed a negative correlation (*p < 0*.*05*) of GLUT4 with HOMA-IRI, and a positive correlation (*p < 0*.*05*) of PTEN with HOMA-IRI at 2 and 4 months in the IUGR offsprings (Fig 4).

### Early catch-up growth is correlated with insulin resistance in the IUGR offsprings

**Fig 5:**
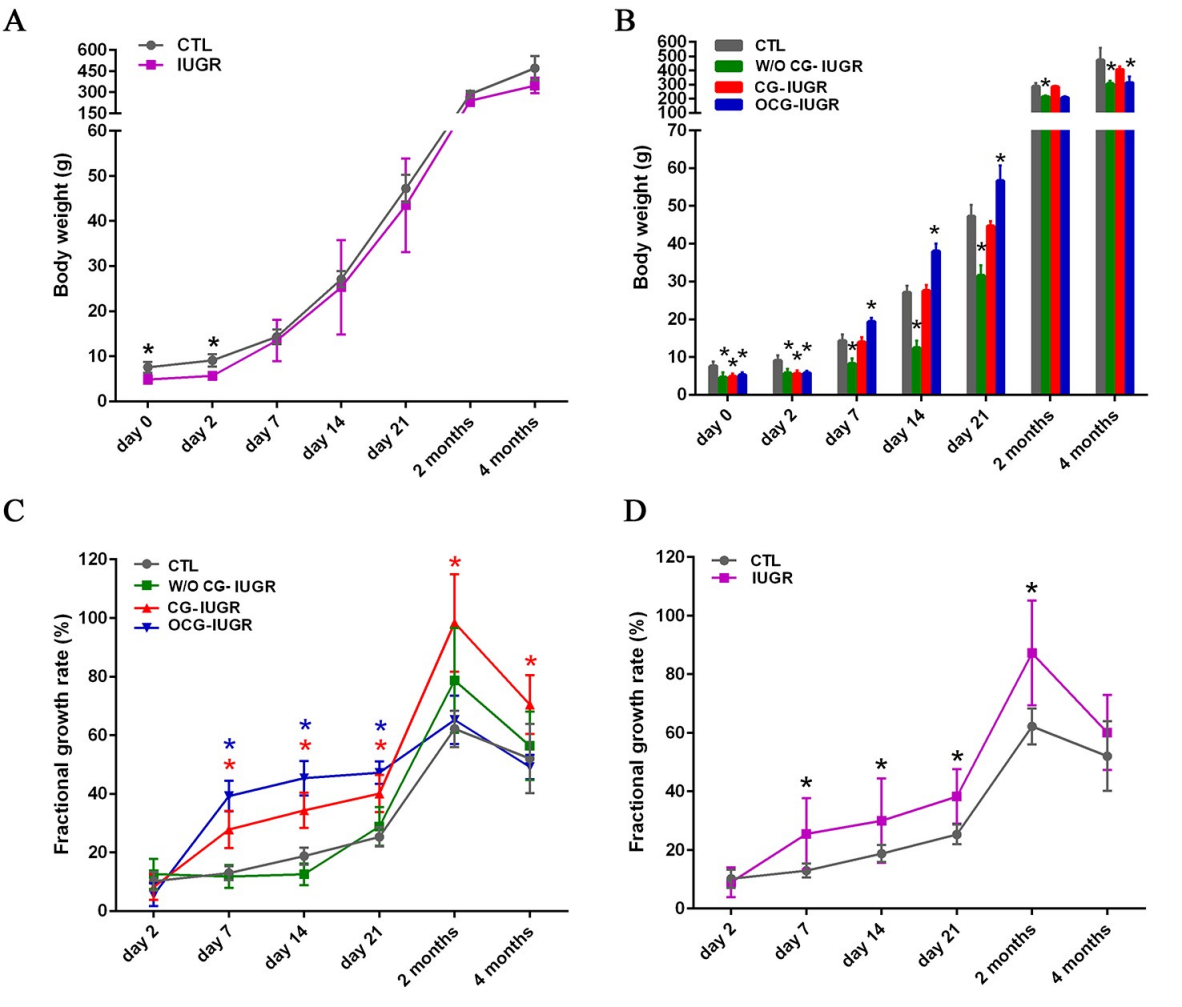
Catch-up growth occurs in the IUGR offsprings. A. Body weights of the controls (CTL) and the IUGR offsprings were recorded at birth (day 0), 2, 7, 14 and 21 days, as well as 2 and 4 months of age. *Data are shown as mean ± SD*. *n = 45;* ^*^p < 0.*05 vs*. *CTL*. B. Based on the mean and standard deviations of body weights, IUGR offsprings were further identified as with catch-up growth (CG-IUGR), with over catch-up growth (OCG-IUGR), and without catch-up growth (W/O CG-IUGR). *Data are shown as mean ± SD*. *n = 45 (CTL)*, *15 (W/O CG-IUGR)*, *18 (CG-IUGR)*, *and 12 (OCG-IUGR);* ^*^p < 0.*05 vs*. *CTL*. C. The fractional growth rate was plotted from day 2 to 4 months for the controls, W/O CG-IUGR, CG-IUGR and OCG-IUGR. *Data are shown as mean ± SD*. *n = 45 (CTL)*, *15 (W/O CG-IUGR)*, *18 (CG-IUGR)*, *and 12 (OCG-IUGR);* ^*^p < 0.*05 vs*. *CTL*. D. The fractional growth rate was plotted from day 2 to 4 months for the controls and the whole IUGR offsprings. *Data are shown as mean ± SD*. *n = 45;* ^*^p < 0.*05 vs*. *CTL*.

Mounting studies have shown that low birthweight is associated with impairs of glucose tolerance and occurrence of insulin resistance, subsequently, type 2 diabetes [34]. Moreover, it has been demonstrated that the accelerated growth that often follows low birthweight is an important risk factor for obesity and type-2 diabetes [18]. In this study, the BWs of IUGR offsprings were recorded and compared. The mean BWs showed no significant differences from 7 days to 4 months of age between the IUGR offsprings and the controls (Fig 5A). Based on the mean and SD of the BWs, the offsprings of IUGR were further classified as CG-IUGR, OCG-IUGR and W/O CG-IUGR, respectively (Fig 5B). We observed 40% (18/45) of IUGR offsprings with CG, 26.7% (12/45) with OCG, and 33.3% (15/45) without CG since day 7. The BWs of W/O CG-IUGR were significantly (*p < 0*.*05*) lower at all time points than those of the controls (Fig 5B). As compared with the controls, the BWs of OCG-IUGR was significantly higher (*p < 0*.*05*) at day 7, 14 and 21 (Fig 5B). Nevertheless, the BWs showed no differences between the controls and CG-IUGR from day 7 to 4 months (Fig 5B). To compare the growth rate, the FGR was calculated and plotted (Fig 5C). The CG-IUGR group had a higher (*p < 0*.*05*) FGR from day 7 to 4 months, while the OCG-IUGR group from day 7 to day 21, in comparison with the controls (Fig 5C). These results suggested a persistent growth in BWs in the CG-IUGR, whereas a transient growth in BWs in the OCG-IUGR. We also compared the FGR between the controls and the whole IUGR offsprings. We observed a significant higher (*p < 0*.*05*) of FGR from day 7 to 2 months in IUGR offsprings compared to the controls (Fig 5D). We thus focused on the FGR that showed significance at the earliest time point day 7, and performed Pearson correlation analysis of the FGR with the HOMA-IRI at 2 and 4 months in the whole IUGR offsprings. Of note, the FGR at day 7 was positively (2 months: *r = 0*.*97*, *p < 0*.*001*; 4 months: *r = 0*.*93*, *p < 0*.*01*) correlated with the HOMA-IRI at 2 and 4 months in the IUGR offsprings.

### Dynamic alterations of the PI3K, PTEN and GLUT4 expressions in skeletal muscle tissues in the IUGR offsprings with early catch-up growth

**Fig 6:**
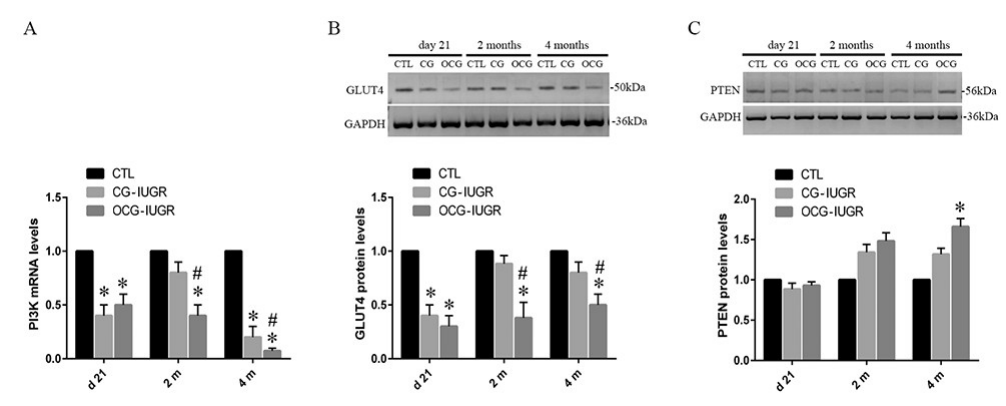
The expression levels of PI3K, PTEN and GLUT4 were analyzed in the IUGR offsprings with early catch-up growth. The mRNA levels of PI3K (A), as well as the protein levels of the GLUT4 (B) and PTEN (C) were assessed and compared among the controls and IUGR offsprings with catch-up (CG) or over catch-up growth (OCG). *Data are shown as mean ± SD*. *n = 15 (CTL)*, *6 (CG-IUGR)*, *and 4 (OCG-IUGR)*. ^*^p <0.*05 vs*. *CTL;* ^*#*^*p <0*.*05 vs*. *IUGR-CG*.

We evaluated the abundances of the PI3K/Akt related signaling at the mRNA and protein levels in the CG-IUGR and the OCG-IUGR rats. The abundances of PI3K at mRNA level and GLUT4 at protein level were significantly reduced (*p < 0*.*05*) at day 21 as well as at 2 and 4 months in both the CG-IUGR and the OCG-IUGR rats in comparison with the controls (Fig 6A and 6B). Of note, the abundances of PI3K and GLUT4 were significantly lower (*p < 0*.*05*) at 2 and 4 months in the OCG-IUGR compared to the CG-IUGR rats. Increases of PTEN protein levels were only detected in the OCG-IUGR rats in comparison with the controls (Fig 6C). Overall, these data suggested a dynamic alterations of PI3K, PTEN and GLUT4 expressions in skeletal muscle tissues in the IUGR offsprings, particularly in the OCG-IUGR rats.

## Discussion

In this study, we showed that the IUGR offsprings with rapid postnatal catch-up growth starting at day 7 was correlated with the occurrence of insulin resistance at 2 and 4 months after birth, in which reductions of the PI3K/Akt related signaling were involved.

As reported by other investigators [14, 15, 18], the rat IUGR model was successfully established via maternal nutritional restriction, which was confirmed by the low birthweights. To assess if insulin resistance was developed in the IUGR offsprings, we measured four glucose metabolic indices. Increased FPG and FINS at 2 and 4 months suggested abnormalities of glucose metabolism and insulin secretion in the IUGR offsprings. The dramatically increased IRI and decreased ISI further demonstrated that insulin resistance occurred in the later development of IUGR offsprings. Insulin acts by increasing the glucose transport rate of each transporter through increasing the number of functional glucose transporters [35]. Insulin resistance in adipose cells and muscle tissue is associated with decreased number and/or activity of GLUT4 [36]. Reductions of muscle tissue GLUT4 protein levels and insulin secretions have been reported in IUGR offsprings [37]. Consistently, we found that the mRNA and protein levels of GLUT4 decreased significantly at all designed time points in the IUGR offsprings. This result revealed a remarkable reduction of glucose transporter GLUT4. However, FPG and ISI at day 21 did not have statistical significance between the IUGR offsprings and the controls. Under the basal state, GLUT4 mainly exists in the membrane-like structure of cells. When insulin stimulates the membrane, GLUT4 rapidly moves to the surface of the membrane to perform transport function [19–22,35,36]. Although the total content of GLUT4 is reduced, the regulation of early body compensatory mechanism and rapid redistribution in the membrane may still make the surface GLUT4 function properly. With the increase of age, the expressions of GLUT4 in skeletal muscle of the IUGR offsprings continued to decrease. With the persistent reduction of GLUT4, the compensatory function of the body has been exceeded the load and cannot continue to maintain normal physiological functions. Thus, obvious insulin resistance appears.

It has been reported that activation of Akt by a PI3K dependent mechanism can stimulate translocation of the GLUT4 [38]. Here, reductions of total PI3K abundances and its phospho-PI3K p85^Tyr458^/p55^Tyr199^ subunit were detected in the IUGR offsprings. The level of phospho-Akt^Ser473^ was also reduced in the IUGR offsprings. These data implied a significant inhibition of the PI3K/Akt signaling activity in the IUGR offsprings. PTEN, as a negative regulator, is responsible for suppression of Akt activation by dephosphorylating phosphoinositide substrates [39,40]. As expected, a significant increase of the PTEN level was detected in the IUGR offsprings. Interestingly, it has been reported that increased PTEN expression is associated with the development of insulin resistance [41]. Loss-of-function PTEN mutations in adipose tissue results in systemic glucose tolerance and improvement of insulin sensitivity because of increased recruitment of the GLUT4 towards the membrane [42]. In glucosamine-induced insulin-resistant skeletal muscle cells, the rate of glucose uptake as well as the expression and translocation of GLUT4 were decreased, whereas the expressions of PTEN and phospho-PTEN were increased [43]. In combination with our findings, it suggests that PTEN may be a promising target for pharmacological interventions aimed at reversing insulin resistance in individuals with IUGR.

Since there is a peroxisome proliferator-activated receptor (PPAR) response element in the promoter region of PTEN, PPARγ acts as a transcriptional factor, having a crucial role on regulating PTEN expression [44]. In inflammatory and tumor-derived cells, PPARγ plays a critical function in regulating PI3K signaling by modulating PTEN level [45]. We analyzed the abundance of PPARγ in nuclear fractions extracted from skeletal muscle tissues, showing that the level of nuclear PPARγ increased dramatically in the IUGR offsprings. These results suggested that PPARγ-mediated increase of PTEN expressions may inhibit the PI3K/Akt activity, leading to reduction of GLUT4 expressions and thus the development of insulin resistance in IUGR offsprings. Pearson’s coefficient analysis also revealed a negative correlation of GLUT4 protein levels with HOMA-IR, and a positive correlation of PTEN protein levels with HOIMA-IR.

Early acceleration of growth that often follows low birthweight is an important risk factor for later development of obesity, type-2 diabetes and cardiovascular diseases [46]. In mice with IUGR, it was found that catch-up growth was related to the occurrence of peripheral and central insulin resistance in adulthood [18]. However, the potential molecular mechanisms by which catch-up growth affect insulin sensitivity remains elusive in individuals with IUGR. In the present study, the BWs were monitored, and the growth rate after birth was presented as the FGR for IUGR offsprings. We found that the mean BWs did not show significant differences from 7 days to 4 months of age between the IUGR offsprings and the controls. However, based on the mean and SD of the BWs, the IUGR offsprings were further classified as CG-IUGR, OCG-IUGR and W/O CG-IUGR starting at day 7. Usually, there are two ways to obtain catch-up growth in IUGR offsprings. IUGR pups were fed normal protein diet after birth; by contrast, IUGR pups were continuously fed low protein diet after birth to restrict catch-up growth [47]. In addition, litter size was reduced in IUGR group. For example, Ye et al. reduced litter size to five pups per litter at birth in the IUGR group, as compared with eight pups per litter in the control group, to ensure the catch-up growth of the offsprings with IUGR [17]. Here, normal diets and water were provided *ad libitum* after birth for the offsprings of IUGR and the control. Both the IUGR and the controls had the same litter size, 5 pups per litter. We observed that some pups with IUGR realized catch-up growth starting at day 7 after birth. The potential reason may be due to differences of food intake. This phenomenon is common in children with IUGR. Nevertheless, the reason why some children or pups with IUGR obtain catch-up growth later in life remains unclear, and should be further investigated in future.

In the current study, CG-IUGR rats persistently presented with a high growth rate starting at day 7, whereas OCG-IUGR rats only had a transiently high growth rate during 7 and 21 days after birth. Although the BWs were lower than the controls at all time points, the FGR of W/O CG-IUGR was similar as that of the controls. This suggested that the IUGR rats without CG can follow growth of the controls. In consistent, the results of FGR by comparing the controls and the whole IUGR offsprings showed that the whole offsprings with IUGR had catch-up growth. At early 7 days of age after birth, catch-up growth was detected in IUGR offspring. Pearson’s coefficient analysis revealed a positive correlation of FGR at day 7 with HOMA-IRI at 2 and 4 months. Thus, it might be a choice to avoid rapid increase of BWs in early childhood. By this, we may reduce the risk for later development of insulin resistance and diabetes. We also compared the level of PI3K, PTEN and GLUT4 between the CG-IUGR and OCG-IUGR rats. Our results showed that the PI3K and GLUT4 abundances were significantly lower in the OCG-IUGR than that in the CG-IUGR rats. In addition, increased PTEN level was detected only in the OCG-IUGR rats. These findings suggested that reduction of GLUT4 potentially mediated by the PTEN/PI3K/Akt signaling may involve the regulation of insulin sensitivity in the IUGR offsprings with catch-up growth. It was reported that mice that underwent IUGR followed by catch-up growth display peripheral and central insulin resistance in adulthood, and that expressions and/or phosphorylation of PI3K p110 subunit and phospho-IRS1 were altered in the arcuate nucleus of the hypothalamus [18]. A prospective study reported that low birthweight, not catch-up growth, was correlated with insulin resistance at 12 months in non-obese infants with small for gestational age status [48]. Thus, the association of the low birthweight in infants with catch-up growth and the development of insulin resistance later in life should be further investigated.

### Conclusions

Taken together, we demonstrated that glucose metabolic abnormalities and insulin resistance were developed in IUGR offsprings, in which reduction of GLUT4 expression possibly mediated by suppression of PI3K/Akt signaling *via* PPARγ-induced PTEN upregulation was involved. Additionally, rats that underwent IUGR followed by catch-up growth was associated with insulin resistance, and also had more dramatic alterations of PTEN, PI3K and GLUT4 expressions, particularly in those with over catch-up growth. Nevertheless, in small for gestational age infants no matter with or without obese, it should be further investigated if insulin resistance is correlated with catch-up growth.
